# The Future of Livestock Management: A Review of Real-Time Portable Sequencing Applied to Livestock

**DOI:** 10.3390/genes11121478

**Published:** 2020-12-09

**Authors:** Harrison J. Lamb, Ben J. Hayes, Loan T. Nguyen, Elizabeth M. Ross

**Affiliations:** Centre for Animal Science, Queensland Alliance for Agriculture and Food Innovation, The University of Queensland, St. Lucia, QLD 4067, Australia; b.hayes@uq.edu.au (B.J.H.); t.nguyen3@uq.edu.au (L.T.N.); e.ross@uq.edu.au (E.M.R.)

**Keywords:** crush-side genotyping, Oxford Nanopore sequencing, MinION, long-read sequencing, livestock, genetics, SNP calling, structural variant, genome assembly

## Abstract

Oxford Nanopore Technologies’ MinION has proven to be a valuable tool within human and microbial genetics. Its capacity to produce long reads in real time has opened up unique applications for portable sequencing. Examples include tracking the recent African swine fever outbreak in China and providing a diagnostic tool for disease in the cassava plant in Eastern Africa. Here we review the current applications of Oxford Nanopore sequencing in livestock, then focus on proposed applications in livestock agriculture for rapid diagnostics, base modification detection, reference genome assembly and genomic prediction. In particular, we propose a future application: ‘crush-side genotyping’ for real-time on-farm genotyping for extensive industries such as northern Australian beef production. An initial in silico experiment to assess the feasibility of crush-side genotyping demonstrated promising results. SNPs were called from simulated Nanopore data, that included the relatively high base call error rate that is characteristic of the data, and calling parameters were varied to understand the feasibility of SNP calling at low coverages in a heterozygous population. With optimised genotype calling parameters, over 85% of the 10,000 simulated SNPs were able to be correctly called with coverages as low as 6×. These results provide preliminary evidence that Oxford Nanopore sequencing has potential to be used for real-time SNP genotyping in extensive livestock operations.

## 1. Introduction

DNA sequencing allows us to examine the underlying genomic information that affects important traits in agriculture. Sequencing technology can broadly be divided into three distinct generations. First-generation technology, such as Sanger sequencing, was characterised by the use of chain termination, second-generation sequencers, such as Illumina, by high-throughput short reads and third-generation sequencers by high-throughput long reads [1,2]. Two technologies have led third-generation sequencing: Oxford Nanopore Technologies (ONT) and Pacific Biosciences (PacBio). A feature of third-generation sequencing technology is that the native DNA is sequenced directly without amplification. This has the advantage of removing the nucleotide biases and alterations in relative abundance of DNA templates that are observed in some short-read sequence data [1,3,4]. Single-molecule sequencing also produces longer reads which can map and characterise complex genomic regions, such as interspersed repeats, with greater specificity. Long reads have greatly improved the de novo assembly of a number of complex genomes. Their ability to span repetitive regions and complex structural variants (SVs) has led to the publication of more contiguous and accurate reference genomes [5,6,7,8]. However, single-molecule sequencing has an associated increase in error rate as a result of not being amplified against a DNA template.

**Figure 1:**
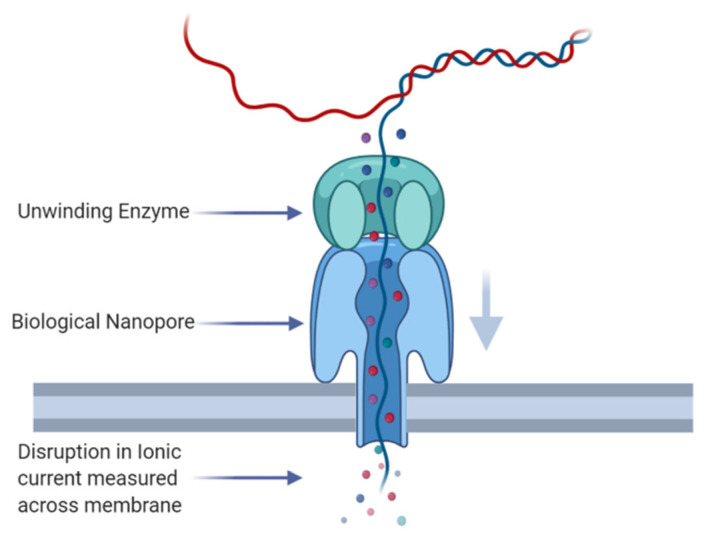
Stylized representation of Nanopore’s sequencing mechanism. Biological nanopores are embedded in a membrane. Double-stranded DNA is enzymatically unwound and the resulting ssDNA passes through the nanopore, disrupting the ionic flow through the nanopore in a characteristic manner dependent on the nucleotide bases within the pore.

ONT sequencing uses a biological nanopore embedded in an electrically resistant membrane [9]. A motor protein, which “unzips” the dsDNA to allow it to pass through the nanopore, is added to the DNA template during library preparation [10]. A current flows through the nanopore, which is disrupted in a characteristic manner by nucleotides passing through the pore (Figure 1, [10]). ONT base calling algorithms use the characteristic disruption in electrical signal by 5 base pair k-mers passing through the pore to interpret the DNA sequence [10]. ONT released the MinION in 2014 [10] and it remains the smallest sequencer on the market at 105 mm × 23 mm × 33 mm [11] while also being powered by the USB port of a computer [10]. Recently, ONT also released the MinION MK1c which combines the MinION sequencer with a high-performance computer for base calling, a high-capacity solid state drive and a touch screen display [12]. The MinION and MinION MK1c are to date the only portable sequencing platforms on the market, a significant advantage over other third-generation sequencing platforms. Some systematic errors remain a problem with ONT sequencing, in particular, the under-representation of homopolymer repeats, caused by an inability for the base caller to identify the breakpoints in signal between homopolymers [13]. However, ONT have continued to release new flow cell chemistry and base calling algorithms to attempt to address this problem. Initially, single-pass accuracies of only 68.4% (Phred score of 5) were reported with the MinION in 2015 [13]. Using the R9.4.1 flow cell chemistry and Guppy base caller, raw read accuracies of 85–90% have been reported [14]. This is considerably less than the 99.999% accuracy in sanger sequencing [15] and 99.92% in Illumina [16]. The release of the R10 flow cell chemistry has reportedly increased the raw read accuracy. However, this has come at a cost to the overall flow cell yield. The error rate in ONT sequencing makes single-nucleotide variant (SNV) and insertion/deletion (INDEL) detection difficult and for this reason it has been largely avoided [17]. Instead, ONT sequencing’s long reads have given it a natural application in the detection of large variants, such as SVs—defined here as deletions, insertions, translocations, duplications and inversions greater than 50 bp [18]—in place of short-read sequencing technologies [17].

ONT have also released a number of other sequencing platforms, which use the same underlying technology. For example, the GridION and PromethION, larger, benchtop cousins to the MinION, provide higher throughput, producing 75–150 Gbp and 2.4–4.3 Tbp, respectively, per run [12,19]. Additionally, the Flongle is a flow cell compatible with both the MinION and GridION which provides a cheaper alternative to traditional MinION flow cells where high yield is not required [12]. ONT has also developed LamPORE, their first diagnostic assay which will be aimed at detecting SARS-CoV-2 [12]. LamPORE is compatible with both the MinION and GridION sequencers and combines barcoded multitarget amplification using a 15 min barcoded library preparation [20]. ONT estimate that LamPORE is capable of processing 96 samples in just over one hour [12]. To complement their line of sequencing platforms, ONT have a number of software packages for raw read base calling (Guppy), data quality control (EPI2ME and Guppy) and analysis (EPI2ME). An android application, F5N, has also been released to allow sequencing and rudimentary data analysis using a smart phone paired with a MinION [21].

The MinION has already been widely used as a tool for rapid diagnostics within human and microbial genetics [22,23,24,25]. Quick, et al. [23] identified, at a species level, a *Salmonella enterica* outbreak in a hospital within 20 min of sequencing on the MinION. However, in another study, the MinION demonstrated that accurate identification of bacteria was possible within 2 h of receiving a DNA sample [22]. Perhaps most impressively the MinION decreased the diagnosis time in a 62-year-old female patient with bacterial sepsis from 6.25 days to just 19 h [26]. The MinION’s portability and ability to base call in real time has also made it popular for tracking disease outbreaks [24,27,28], including recent outbreaks of ebola [24], zika [28] and COVID-19 [29,30] as well as more isolated outbreaks of human metapneumovirus [31] and the fungal pathogen *Candida auris* [32].

Here we review a number of current applications of the MinION such as rapid diagnostics and de novo genome assembly and discuss their potential applications to the livestock industry, taking lessons from medical research. We discuss and review the potential for the MinION as a rapid diagnostic tool for diseases relating to pigs, chickens and cattle as well as benefits associated with using the MinION to genotype causative mutations including structural variants for genomic prediction. In particular, we present a future application in “crush-side genotyping”: the rapid, on-farm low-coverage genotyping of cattle for genomic prediction.

## 2. Current and Future Applications in Livestock

Within the field of agriculture, the uptake of Nanopore sequencing has been relatively slow by comparison to medical research, and slower still within livestock. The applications of the sequencer within livestock agriculture have largely been extensions of disease tracing and rapid diagnostics.

### 2.1. Rapid Diagnostics of Pathogens in Livestock

African swine fever virus is the causative agent behind the 2019 global outbreak of African Swine Fever (ASF), a highly contagious disease endemic to several sub-Saharan African countries [33]. ASF has up to a 100% mortality rate [34], and modelling of the economic impacts of the disease indicate that pork prices could increase by 17–85% [35] due to the current outbreak which has put an enormous strain on global pork production [36]. Early and rapid diagnosis of ASF is a crucial step in controlling the impact of the disease given the absence of a vaccine [37]. Results from current diagnosis methods may take several days or even weeks due to significant logistical and economic constraints [37]. O’Donnell, et al. [38] demonstrated the potential ability for the MinION to provide rapid diagnosis of this virus with the development of the African swine fever fast analysis sequencing tool (ASF-FAST). ASF-FAST monitors the designated sequencing output directory for .fastq files [38]. New .fastq files are transferred into a “Que” folder where reads are separated by barcode using Porechop [39] and processed using the Burrows–Wheeler alignment tool [40] to perform a reference-guided assembly [38]. ASF-FAST then performs a local BLASTN of the consensus sequence against known ASFV reference strains [38]. An output report with summary statistics in a PDF is then sent by SMS upon request [38]. Sufficient viral sequence was captured for complete resolution of the 192 kb genome [41] within 10 min of sequencing using ASF-FAST [38]. Accurate characterisation and diagnosis of porcine viral enteric disease in piglets has also been demonstrated using the MinION [42]. The ASF-FAST pipeline demonstrates the benefits in the MinION’s real-time sequencing ability, and could be expanded to meet other diagnostic needs within livestock agriculture.

Avian cholera caused by the bacterium *Pasteurella multocida* has significant economic impacts in the poultry industry, principally in free range operations [43]. It has had a particularly severe impact on the Australian poultry industry with a number of outbreaks and subsequent re-infections [43]. Whole killed cell vaccines are currently used to prevent outbreaks. However, these only provide protection against strains with identical or near identical lipopolysaccharide (LPS) structure [43,44]. The MinION could be used to not only provide rapid diagnosis of diseases such as fowl cholera in poultry flocks but also provide real-time characterisation of the LPS structure for accurate targeted vaccination.

Bovine respiratory disease (BRD) is the most common cause of mortality of cattle in North American and Australian feedlots [45]. In North America alone BRD is estimated to cost the agriculture industry over $1 billion USD [46]. BRD is typically induced by a combination of stress factors as well as exposure to a range of infectious agents [47,48]. Rapid diagnosis of BRD and characterisation of the causative agent is a key mitigation strategy for disease control in feedlots [49]. The MinION is capable of identifying the viruses responsible for BRD using Oxford Nanopore’s rapid sequencing kit [50,51]. McCabe, et al. [50] reported that library preparation was complete in approximately 10 min and enough reads were produced in the first hour of sequencing to correctly identify three viruses present in the sample. Moreover, they also concluded the MinION has potential for the rapid diagnosis of viruses in regional veterinary settings [50]. The cassava virus action project has already demonstrated that the MinION can be used for rapid diagnostics in agriculture in the field where laboratory access is limited [52]. Live export is another area in which the MinION’s ability to rapidly diagnose a range of diseases could be exploited to improve animal welfare. For example, rapid sequencing diagnostics could be applied to animals before they enter the live export supply chain or a feedlot, to prevent disease outbreaks or identify animals not fit to travel.

In agriculture, the emergence of antibiotic resistant bacterial strains is of significant concern. Strains of multidrug-resistant *Mannheimia haemolytica* have already been identified in cattle with BRD using the MinION [53]. The MinION could reportedly predict resistance to β-lactams, tetracyclines, lincosamides, phenicols, aminoglycosides, sulfonamides and macrolides [53]. An immediately evident application for such abilities is mastitis in dairy cattle, where antibiotic resistant genes within an infection could be diagnosed within hours. Effectively targeting treatments to the specific bacterial infection has the potential to both reduce the treatment cost as well as improve veterinary outcomes by saving needless use of broad-spectrum antibiotics and increasing the efficacy of treatment.

Other real-time analysis tools have also been developed for real-time mapping and coverage statistics. The Read Assignment, Mapping and Phylogenetic Analysis in Real Time (RAMPART) tool [54] runs concurrently with ONT’s MinKNOW to provide a real-time graphical overview of genome coverage and reference matching of reads as they are base called and mapped by MinKNOW [54]. RAMPART has been adopted by the ARTIC network project which aims to develop an end-to-end system for tracking viral outbreaks using real-time epidemiological information [55]. The ARTIC network pipeline is a user-friendly bioinformatics pipeline that combines reference alignment, post-processing, variant calling and consensus building using a number of popular Nanopore-compatible tools [55]. Despite being initially developed for use in human epidemiology, reference genomes for strains of any number of economically important livestock pathogens could be used with RAMPART to monitor sequencing depth in real time for rapid diagnosis.

A current limitation of viral detection using ONT is the titre of the viral particle. This is because the likelihood of sequencing viral DNA compared to that of the host genome is directly related to the proportion of viral DNA in the sample. Because viral genomes are many orders of magnitude smaller than mammalian genomes, there needs to be many orders of magnitude more viral genomes in the sample than host genomes [50]. One possible solution to this limitation is the upcoming release of adaptive sequencing by ONT [56]. Adaptive or read-until sequencing describes the selective sequencing of particular genomic regions by reversing the flow of ions through specific nanopores [56,57]. Through this, non-target DNA molecules can be ejected from the nanopore increasing the number of available pores for target sequences. This allows enrichment of regions to a desired coverage without additional library preparation procedures [56,57]. By using graphics processing unit (GPU)-compatible base calling, sequences can be base called in real time and either rejected or allowed to continue sequencing [56]. Another approach by Kovaka, et al. [57] involves the targeting of k-mers with an estimated similar electrical signal to the desired enrichment regions. This eliminates the need for GPU base calling which could potentially lend itself towards regional applications of the MinION with adaptive sequencing. As a result, adaptive sequencing could be used to target viral diseases with significant economic effects on the livestock industry, including swine flu, foot-and-mouth, brucellosis, tuberculosis and bovine viral diarrhoea virus [58,59,60]. To date, these protocols have only been applied to medical research. However, they hold great promise for the realisation of in-field diagnostics.

Pipelines such as RAMPART and ASF-FAST demonstrate the proven ability for the MinION to significantly decrease diagnostic times and increase treatment specificity. Applications from human medical research can be applied directly to agricultural diagnostics. Importantly, the MinION not only presents an opportunity to lower disease burden, but also provides a mechanism for the reduction in broad-spectrum antibiotic treatment in agriculture. Currently, the high sequencing error rate as well as the relatively higher cost per sequencing run are two hurdles on the path to the wider adoption of ONT sequencing in agriculture. However, it is hoped that the release of adaptive sequencing will address these issues.

### 2.2. Reference Genomes

Reference genome assemblies are essential in understanding an organism’s genetic diversity. Genome assemblies allow us to characterise genetic variants and also reduce the computation time required to analyse sequence data [61]. Through characterising genetic variants, they have allowed us to better understand genetic diseases and in agriculture, in particular, to leverage heritability for accelerated genetic gain. In livestock, genome assemblies exist for all of the major domesticated species: cattle [62], chicken [63], sheep [64], goats [61] and pigs [65]. A number of sequencing platforms have been used to develop reference genomes, each with their respective advantages and disadvantages (Table 1). Historically, reference assemblies were created using short-read sequencing technology and were less accurate than what can be achieved now with long-read technologies. An estimated 14.4 Mbp of the chicken (*Gallus gallus*) reference assembly was discovered to be misassembled contigs; while in the bovine (*Bos taurus*) reference assembly, 39 Mbp was estimated to be misassembled contigs [66].

**Figure 2:**
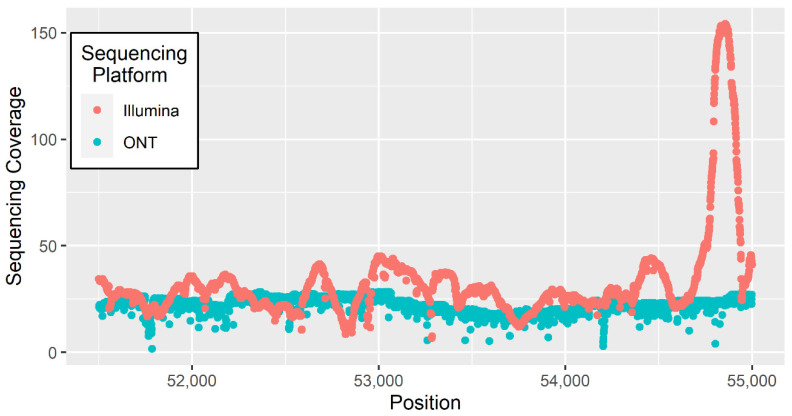
Sequencing coverage across a 3000 bp window for Illumina and ONT sequencing data aligned to the *B. taurus* reference genome [76].

ONT sequencing’s unparalleled read length has allowed for the assembly of a number of complex genomes such as the protozoan parasite *Trypanosoma cruzi* [7] and the mountain-spinach *Atriplex hortensis* [8]. The ability to sequence complex genomes is in part due to long reads being able to span large repetitive regions which second-generation sequencing technologies typically fail to accurately assemble. The inability for short reads to map complex regions results in uneven sequencing coverage in comparison to long reads (Figure 2). A comparison of two genome assemblies using Nanopore and PacBio sequencing in rice found the Nanopore assembly to be more contiguous, having only 18 contigs [67], whereas the PacBio assembly featured 394 contigs [67]. The Nanopore assembly also reportedly had fewer assembly errors caused by long repetitive regions [67]. The individual base accuracy of the ONT assembly, however, was lower than the PacBio assembly, despite polishing the ONT assembly with 70 × Illumina short reads [67]. Single-nucleotide errors in the ONT assembly formed clusters of which 94% were in complex genomic regions and therefore had Illumina short-read coverage of <5 × [67]. This highlights the importance of selecting an appropriate sequencing platform to meet the desired level of assembly contiguity and individual base accuracy.

Relatively high sequencing coverage is necessary for accurate assemblies when using Nanopore data as a result of the high sequencing error rate. For small genomes, this is easily achieved. However, for larger genomes, the higher cost per Gb for Nanopore sequencing means cheaper, highly accurate platforms such as Illumina are now used alongside Nanopore data, such as in the case of the snow sheep *(Ovis nivicola)* genome assembly [68]. This decreases the cost of sequencing and increases the individual base accuracy of genome assemblies. Reference genomes for the endangered giant redwood (*Sequoia sempervirens*) and giant sequoia (*Sequoiadendron giganteum*), both trees native to California, were also created using Illumina and Nanopore data [69]. These genomes were 8.2 and 26.5 Gbp, respectively, demonstrating an ability for the MinION to be used for very large genomes [69]. The giant sequoia’s reference genome also features the longest scaffolds assembled in any organism to date with the largest being 985 Mbp and the scaffold N50 being 690.55 Mb [69].

**Table 1:** Comparison of bench top second-generation sequencers as well as some of PacBio’s and ONT’s platforms.

Sequencing Platform	Read Length	Data Output	Run Time	Single-Pass Error Rate (%)	Reference
Illumina Hiseq 2000	2 × 150 bp	150–200 Gb	1–6 days	0.16	[70,71]
Illumina NextSeq 550	2 × 150 bp	120 Gb	15–18 h	0.16	[70,71]
Pacbio RS II	10–15 kb on average	0.5–1 Gb	0.5–4 h	10	[16,71]
PacBio Sequel	10–15 kb on average	15 Gb	Up to 20 h	10	[72]
PacBio Sequel II	10–15 kb on average	80–100 Gb	Up to 30 h	10	[73]
Oxford Nanopore MinION	10–20 kb on average	20–30 Gb (per flow cell) Up to 1 flow cell/run	Up to 96 h	5–20	[12,74]
Oxford Nanopore GridION	10–20 kb on average	20–30 Gb (per flow cell) Up to 5 flow cells/run	Up to 96 h	5–20	[12,74]
Oxford Nanopore PromethION	10–20 kb on average	100–180 Gb (per flow cell) Up to 48 flow cells/run	Up to 96 h	5–20	[12,74]

ONT’s ability to characterise complex genomic regions has the potential to increase the accuracy of reference genome assemblies in livestock. The ability of all three major ONT sequencing platforms: the MinION, PromethION and GridION, to facilitate accurate genome assemblies may help identify targets for future breeding and selection. More accurate assemblies will also facilitate the incorporation of more complex genomic variants into genomic prediction and increase the power of genomic variant association studies [75].

### 2.3. Structural Variants

Beyond applications in improving the accuracy of reference genomes and rapid diagnostics, the unique mixture of read length and real-time data acquisition leans towards potential uses in calling genomic variants in real time. In particular, the MinION lends itself towards accurate SV characterisation and calling, which has already been demonstrated in humans [77,78]. SVs are estimated to contribute between 0.5 and 1% of genomic differences between individuals while comparatively single-nucleotide variations contribute only 0.1% [18]. This highlights the potential genetic variation that, for the most part, is poorly characterised to date, particularly in species other than model organisms and humans.

The proportion of genetic variation explained by SNP markers is often referred to as $h_{m}^{2}$, while variance explained by genome-wide-significant SNPs is referred to as $h^{2}{}_{GWS}$ [79]. Heritability studies within families reveal a difference between total additive genetic variance ($h^{2})$ and $h^{2}{}_{GWS}$ which is referred to as “missing heritability” [79]. Ultimately these studies reveal $h^{2}{}_{GWS}<\;h_{m}^{2}<h^{2}$ [79], which is hypothesised to be the result of genomic variants that are not well tagged by SNPs [79]. Even traits such as fertility in dairy cattle, which could be assumed to be under selective pressure with reasonable confidence, have some missing heritability [80]. A possible source of this missing heritability is larger genomic variants such as SVs [81,82]. SVs range in size from tens to millions of base pairs [83] or even entire chromosomal restructuring [84]. This means that the possible genetic variation explained by SVs may be magnitudes more than that of single-nucleotide variants.

SVs, such as SNPs, can alter the gene itself [85], but may also affect the enhancer or promoter or change its proximity to the gene [86]. The best understood mechanism through which SVs may alter phenotypes is by copy number alteration of a dosage sensitive gene or cluster of genes [86,87,88], such is the case in Williams–Beuren syndrome [89], Smith–Magenis syndrome [90] and systemic lupus erythematosus [91]. SVs have also been found to alter 3D chromatin structure [92,93]. Large deletions and inversions demonstrate detectable effects on chromatin contacts; however, we do not yet understand the impacts of these alterations on an individual’s phenotype [92]. Significant work has been carried out in human genetics to ascertain relationships between a number of complex diseases and SVs [94,95,96]. For example, the age of onset of Alzheimer’s disease is poorly modelled by SNPs alone, but with consideration of structural variants [97,98], the age of onset of Alzheimer’s disease can now be accurately predicted in over 90% of the at-risk population [98]. Similarly, it is hypothesised that SVs are accountable for missing heritability in sporadic amyotrophic lateral sclerosis [81]. Generally speaking the mechanistic and molecular links between SVs and phenotypes are still poorly understood, particularly outside of humans and model organisms [18]. More accurate characterisation of SVs may help to clarify these links.

Short-read technologies struggle to identify SVs, in particular copy number variants in comparison to the MinION. In 14 *Drosophila melanogaster* genome assemblies, more than 20,000 SVs were identified by long-read sequencing of which almost 40% were reportedly undetectable using short-read sequencing [99]. The SV allele frequency in the *D. melanogaster* population relative to amino acid polymorphisms also suggested that SVs are more likely to impact phenotype than non-synonymous SNPs [99] making them candidates for being rare alleles with large phenotypic effect [99]. This highlights the potential power of using long-read sequencing platforms such as the MinION to better characterise SVs.

Voichek, et al. [100] compared the correlation between SNPs and phenotypic traits to the correlation between larger genomic variants such as insertions, deletions and rearrangements to phenotypic traits in a genome wide association study. They developed a range of k-mers that represented structural variations and tested the association between the k-mers and desirable phenotypes in *Arabidopsis thaliana*, tomato and maize [100]. K-mers typically had stronger statistical association with phenotype than SNPs and a number of new associations were found exclusively using k-mers [100]. K-mers have the potential to reveal new alleles associated with desirable phenotypic traits in maize [100]. However, the short-read sequencing which was used produced a high fraction of non-uniquely mapped reads in the maize population [100]; a challenge that would likely be overcome using long Nanopore reads [101].

In livestock, there are only a handful of well-characterised structural variants with known economically significant effects. A 10 bp duplication in the *Pax7* gene is associated with increased body weight, body height and hip width in a number of Chinese cattle breeds [102]. Another phenotype of economic importance in agriculture caused by an SV is the poll phenotype in cattle. The poll phenotype, a desirable trait in both the dairy and beef industries, describes a lack of horns in cattle [103,104,105]. It is controlled at the poll locus on bovine chromosome one [105,106] by at least three known autosomal dominant poll (P) alleles [107,108]. All three alleles are SVs ranging in size from a 212 bp duplication replacing 10 bp [108] to an 80 kb duplication [109]. The poll phenotype is an example of well-characterised SVs with both economic and animal welfare importance in agriculture [109]. Being a highly desirable Mendelian dominant trait, homozygous poll (PP) animals are of greater value than heterozygous (Pp) cattle. This makes it an ideal candidate for real-time calling as there is no phenotypic way to distinguish poll homozygotes from heterozygotes. Recently, another example of an economically important SV was characterised in sheep using Nanopore sequencing [110]. The SV, a 7.9 kb deletion was found to be responsible for a novel *BCO2/enJSRV* hybrid protein that was found to cause undesirable yellow discolouration of adipose tissue [110]. A genetic test for this trait will allow on-farm management decisions before the trait is detected at time of slaughter. A number of SVs linked to coat colour have also been characterised in cattle [111,112], while of arguably less economic significance the phenotypes caused by these SVs may be of interest to stud breeders. Two complex SVs involving the *KIT* locus (*B. taurus* chromosome 6) have been found to cause colour sidedness in a number of European breeds [111,113]. A quadruplication of a 6 kb region upstream of the *TWIST2* gene in cattle is strongly associated with a second coat-related phenotype: an area of unpigmented skin and coat around the animals’ mid-section [112,114]. These studies highlight both the additional predictive power that may be leveraged using larger genomic variants but also the suitability of the MinION sequencer to this application [115]. This application could be harnessed further by using the MinION to incorporate causative mutation (particularly SV) calling into genomic selection.

### 2.4. DNA/RNA Modification

Another benefit of ONT sequencing is the ability to directly sequence both DNA and RNA molecules to identify molecule modification. Over 17 and 160 post-transcriptional modifications have been characterised in DNA and RNA, respectively [116], with the most common being methylation [117]. These modifications influence DNA-protein interactions and play a crucial role in biological development [118] and ageing [119,120]. Bisulfite sequencing is generally regarded as the gold standard for DNA methylation profiling [121,122]. However, bisulfite DNA reads often have a lower mapping rate, uneven genome coverage and sequence biases [116]. Alternatively, methylated DNA immunoprecipitation can also be used for methylation profiling. However, this method does not provide base resolution information [123]. RNA modifications are generally also captured using immunoprecipitation alongside reverse transcription and sequencing [123]. However, these processes often only interrogate a specific group of modifications at any one time [116]. The MinION’s ability to sequence DNA and native RNA means it can deliver sequence information as well as post-transcriptional modification profiling. In the case of DNA sequencing, this means methylation profiling can be performed alongside genotyping, which could be incorporated into pipelines for predicting predisposition to premature ageing [124,125], disease [126] or desirable phenotypic traits [127]. Methylated CpG sites have also been demonstrated to be a significant contributor to Nanopore SNP errors and masking of methylation regions has been shown to decrease the error rate of Nanopore sequencing [25], using this method SNP calling accuracy using the MinION could be increased.

One example where understanding the importance of methylation on a trait for economic benefit might be ossification. Ossification is a measure of physiological age of a beef carcass and extensive ossification is an undesirable quality in beef cattle. Ossification is associated with increased dark cutting which is the discolouration of meat as a result of high pH post-mortem [128]. Dark cutting results in a shorter shelf life and bland flavour [129] and therefore producers are often penalised $0.5 AUD /kg of carcass weight [128]. With a strong correlation between ageing and methylation already established in humans [130,131,132], methylation markers for pre-mature ageing and ossification could be developed and called using the MinION. Evidence also suggests that methylation may play a role in stress-related psychiatric disorders and stress-induced depressive behaviour [133,134]. Therefore, methylation markers for livestock welfare and temperament could also be called using the MinION.

### 2.5. Genomic Prediction and Crush-Side Genotyping

Genomic prediction, initially proposed by Meuwissen, et al. [135], has been extensively used within a number of livestock industries such as poultry and dairy. Current genomic prediction typically uses SNP arrays to genotype a large number of markers throughout the genome. These markers are used to calculate the genomic relatedness of animals in a reference population with known phenotypes [135]. By comparing the genomic relatedness of animals with unknown phenotypes to those in the reference population, a genomic estimated breeding value (GEBV) can be calculated [135]. This has allowed some intensive agricultural industries to transition from purely phenotypic trait selection to genomic selection (provided large reference populations of genotyped and phenotyped individuals are established [136]).

A number of limitations to using SNP arrays for genomic prediction exist [136]. A key economic limitation of SNP arrays is that the larger or denser a SNP array, the more expensive it becomes [137]. A further limitation to SNP array genotyping is the inability to accurately genotype SNPs whose flanking regions align to multiple regions in the genome. As an example, it is common practice to remove between 30 and 40 k SNPs from the Brassica 60 k SNP array [138]. Furthermore, SNPs on SNP arrays are typically only selected if both alleles are common and therefore cannot be in complete linkage disequilibrium (LD) with a causal variant with one rare allele [79]. Therefore, SNPs discovered using SNP markers in a GWAS are unlikely to account for all genetic variation as rare variants likely will not be tagged by the genotyped SNPs [79]. The MinION’s ability to generate whole genome sequence data could mean, with sufficient computing power, both common and rare SNPs as well as structural variants could be called, decreasing the missing heritability in genomic prediction. However, the achievable accuracy of genomic predictions given the high error rate of Nanopore sequencing must be investigated before this application can be adopted.

**Figure 3:**
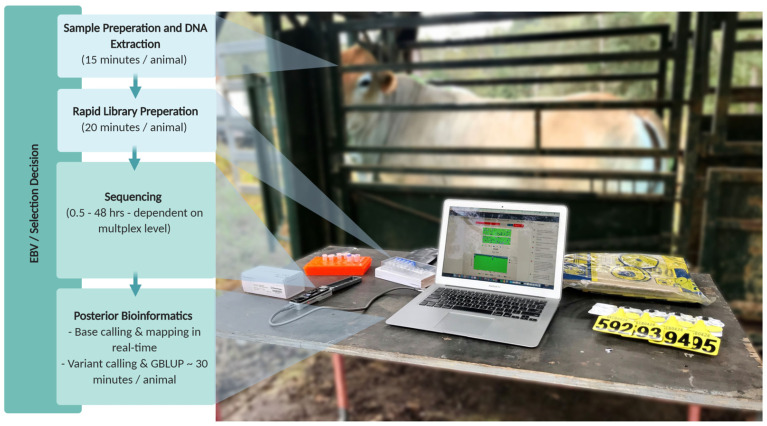
On-site genotyping of a brahman heifer in Queensland using the MinION. With multiplexing a mob of cattle could be genotyped overnight using this pipeline and selection decisions made the following morning using genomic breeding values.

Outside the biological limitations of SNP genotyping by array, this method is also impractical in some industries. For example, in Australia’s northern beef industry, cattle are often only handled once a year, in a mechanical restraint commonly referred to as a ‘crush’. Additionally, northern Australian cattle enterprises will typically only make a mail ‘run’ once or twice a fortnight. This makes proximity to laboratories and turnaround time significant limitations to implementing genomic selection in practice. To address this need, we propose the future application of the MinION sequencer to rapidly on-farm genotype cattle for genomic selection in Australia’s northern beef industry, which we term “crush-side genotyping” (Figure 3). Such a method would allow point of management decisions such as culling and young bull selection to be informed by GEBV, in industries where laboratory turnaround time has limited the use of SNP array genotyping. For example, groups of cattle held in a holding yard could be genotyped overnight. This would allow for management decisions to be made the following morning, rather than weeks later, by which time the cattle would be released and not seen until the following year. Using the MinION, whole genome sequence data could be imputed from SNPs, INDELS or copy number variants (CNVs) which are reliably called for each animal. This would require additional computation, but could be highly parallelised. The concept is similar to that proposed by Ros-Freixedes, et al. [139], where very low sequence coverage is used to genotype animals. A method similar to hybrid peeling, a technique for pedigree-based imputation [140,141], could also be incorporated for fast imputation, phasing and calling. Li, et al. [142] demonstrated that low-coverage (0.5–1 ×) short-read, second-generation sequencing, can increase the power of GWAS and decrease the measurement error of polygenic risk scores for coronary artery disease in humans when compared to SNP arrays. Although these results were reported using highly accurate second-generation sequencing, the ability for long reads to map more accurately would likely increase the accuracy of phasing haplotypes at low coverage. The use of the MinION would additionally allow the incorporation of larger genomic variant markers or causative mutations into genomic selection for increased accuracy.

To date, true real-time crush-side genotyping is not feasible as two major limitations exist: cost and the development of a rapid wet-lab pipeline. The pipeline turnaround time is being actively addressed by a number of groups globally. The application of the MinION in disease outbreaks has driven development of rapid protocols for DNA extraction [143], in-field computing [144] and library preparation [145]. For example, Boykin, et al. [146] have demonstrated the PDQeX nucleic acid extractor (MicroGEM, Dunedin, New Zealand), which is capable of extracting DNA from up to 24 different tissue samples in less than 20 min, can rapidly extract DNA for use with the MinION. Additionally, Zou, et al. [143] have developed a cellulose paper-based DNA purification technique that is able to extract and purify DNA in 30 s, another example of available rapid DNA extraction methods. Another development that could increase the speed of the wet-lab turnaround time is ONT’s VolTRAX [12], which completely automates the library preparation and flow cell loading steps. Ideally the VolTRAX could be combined with Nanopore’s field sequencing library preparation kit. The field sequencing kit is capable of library preparation in 10 min and requires no cold storage and limited laboratory equipment [12], although this protocol currently produces a lower sequencing yield. The VolTRAX and rapid sequencing kit will increase the reproducibility of sequencing runs and decrease the library preparation requirements and turnaround time. The release of the MinION Mk1c has also addressed the hurdle of infield sequence acquisition with its GPU base calling ability and 1 TB solid state drive (SSD) built in with the sequencer. The transition towards GPU-compatible base calling packages has shortened the analysis turnaround time by an order of magnitude [147]. Base calling speeds have increased from 120,000 bases/s using a central processing unit (CPU) in 2017 to over 1,000,000 bases/second using ONT’s GPU basecaller Guppy [148]. A lack of internet connection in remote locations presents an obvious hurdle to crush-side genotyping analysis. However, an offline metagenomics analysis tool, MINDS (MinION Detection Software), has already demonstrated that offline analysis of MinION data is currently possible [149]. From 173,000 reads, MINDS correctly identified 19 of 20 species from the MSA-2002 mock metagenomic community in 15 min, without internet connection [149]. Although, this clearly demonstrates the current ability to efficiently analyse MinION data while offline, the significant increase in data throughput necessary in the context of crush-side genotyping means that considerable optimisation would be required.

The second key limitation, cost, will likely be addressed by parallelising the sequencing. Currently, SNP genotyping is available in a variety of densities, from ultra-low ~ 200 SNP parentage arrays to high density 777 k, where density refers to the number of SNPs genotyped and therefore the degree of separation between markers. Traditionally, the beef industry, in particular Australia’s Northern beef industry, use commercial 50 k SNP arrays to achieve acceptable genomic prediction accuracies. These 50 k SNP arrays today cost $35–55 AUD [150], a significant drop from $400–500 AUD per animal for 10,000 SNPs in 2006 [151]. For crush-side genotyping to be feasible, the cost per sequencing run will not necessarily have to approach the cost of SNP array genotyping. Instead, in industries where SNP array genotyping has not been used due to the limitations discussed, it will only need to cost less than the increase in profitability resulting from genomic selection. Peñagaricano [152] estimated genomic selection has resulted in the doubling of annual genetic gain for economically important traits in dairy cattle since its implementation. Most notably, a 3-fold to 4-fold increase in genetic gain for traits that can only be measured late in life, such as female fertility, have been reported [152]. Currently, a single MinION sequencing run costs ~$700 USD [12] and can consistently yield over 20 Gb using R9.4 flow cells [12]. This yield has increased dramatically from less than 1 Gb in 2014 using ONT’s R6.0 flow cells [153]. Alongside the increase in sequencing yield, in the last six years, the single-pass error rate from Nanopore sequencing has decreased almost 4-fold from 38.2% using the R6.0 flow cells [154] to 9–10% using R9.4 flow cells [155].

We see the possible introduction of adaptive sequencing as a key link in the chain to significantly reduce the cost of sequencing and the required whole genome coverage. A number of markers spread evenly throughout the genome, similar to SNP array genotyping, could theoretically be enriched in real time using adaptive sequencing. This would produce accurate marker genotypes at comparatively ultra-low whole genome coverages. Another method that could be used for rapid selective enrichment is LamPORE’s loop-mediated isothermal amplification (LAMP). LamPORE uses a 35 min LAMP to amplify three highly conserved genes in SARS-CoV-2 for rapid diagnosis [12]. The LAMP method has been optimised to be highly parallelised (9000 samples can be barcoded in 24 h) [12], which is very attractive for crush-side genotyping. However, the additional wet-lab steps are currently also a limitation of this technique. The affordable characterisation of rumen microbiome and rapid diagnosis of pathogens in livestock could be more suitable applications of this method. Alternatively, CRISPR-Cas9-targeted enrichment is another method in ONT’s toolkit which could be used in place of adaptive sequencing [156]. Currently, CRISPR-Cas9-targeted enrichment provides greater enrichment of desired loci than adaptive sequencing [56,156,157], but introduces additional wet-lab steps [156]. CRISPR-Cas9 could potentially be used where relatively few enriched loci are required, for example in parentage verification or the targeted introduction/eradication of specific genotypes (e.g., deleterious mutations) in a herd.

This method of enrichment shares similarities with genotype by sequencing (GBS), a cost-effective method for low-coverage genotyping [158,159,160]. GBS uses restriction enzyme cleavage to reduce genome complexity for sequencing on short-read sequencing platforms [158,159,160]. This method of low-coverage sequencing has the advantage of not requiring a priori: a reference genome in the case of adaptive sequencing and CRISPR-Cas9 enrichment [161]. This means that GBS can simultaneously be used for variant discovery as well as genotyping [158]. Additionally this method is currently more cost effective than the proposed crush-side genotyping. However, a number of limitations to GBS exist. Most importantly, the second-generation sequencing platforms used for GBS are currently not portable, which is the major drawkback crush-side genotyping aims to solve. The real-time nature of adaptive sequencing also means that the chosen enriched loci can be changed during the sequencing run [56]. Enrichment could also be turned off once sufficient sequencing coverage is achieved to capture genome wide variants.

Targeted sequencing enrichment on the MinION would allow for the sequencing of multiple samples per flow cell. This could be combined with ONT’s barcoding library kits. Up to 96 samples can currently be multiplexed using ONT’s PCR barcoding kit. However, this requires an additional 60 min library preparation and PCR. The rapid barcoding kit (10 min library preparation) currently only uses 12 barcodes, but would likely be more suitable to ensure the pipeline remains rapid and portable [12]. It is likely that the number of samples that can be barcoded with the rapid kit will increase over time. If 6 × coverage is assumed to be the minimum requirement for accurate genotype calling on the majority of loci using the MinION and an average of 50,000 SNP markers are needed for accurate genomic prediction then—given reported sequencing enrichment rates [56,156,157]—we estimate that 1.8 Gb will be necessary per animal. We assume the maximum theoretical yield from a single MinION flow cell is 20 Gb. MinION sequencing of cattle tail hair DNA has yielded up to 45 Gb per flow cell using non-targeted sequencing. Based on these assumptions, multiplexing 11 animals per flow cell is possible. At this multiplexing level, the sequencing cost per animal is approximately $90 USD. Additionally, minimum coverages for accurate genotyping may decrease with improvements in sequencing accuracy and software advances. Furthermore, accurate genomic prediction is regularly achieved with as little as 4000 SNP markers in cattle, which would decrease the price further. Crush-side genotyping, may not yet be feasible and relies heavily on emerging technology. However, we believe the rapid development of the technology means crush-side genotyping will be feasible in the short to mid-term.

One limitation to the current application of crush-side genotyping is reduced flow cell yields when using enrichment methods. Payne, et al. [56] reported a significant drop in sequencing efficiency while using adaptive sequencing: markedly lower enrichment was achieved towards the end of the sequencing run. This was attributed to the degradation and blocking of pores [56]. Payne, et al. [56] overcame this issue by washing the flow cells with nuclease. Further optimisation of these recently published methods: adaptive sequencing, Cas9 enrichment and LAMP sequencing, will see the feasibility of crush-side genotyping increase shortly.

The ability of the MinION to call SNPs at known loci at low coverage in *Brassica napus* L. has already been assessed [138]. Despite having higher error rates than other sequencing platforms, the MinION achieved an accuracy of 96% in calling SNPs at four million known locations [138]. However, a predominantly homozygous canola population was used in this study, which would result in a higher accuracy than would be expected in outbred populations [138]. However, the ability for the MinION to accurately genotype SNPs in a heterozygous population at low coverage has not been investigated. Therefore, we conducted a preliminary investigation into the feasibility of crush-side genotyping using simulated Nanopore read data to assess the accuracy of SNP calling in heterozygous populations.

## 3. Materials and Methods

We conducted a simulation study to test the hypothesis that SNP genotypes can be accurately identified in a diploid species using MinION data; and that changing the filtering and calling parameters used to call SNPs will affect the accuracy of the identified SNP.

**Figure 4:**
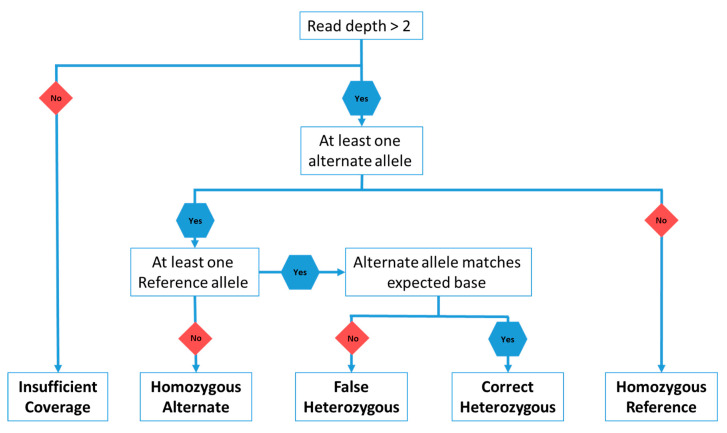
Flow chart for the genotype calling script that was used with the VCF files generated by Samtools and BCFtools.

An artificial diploid of chromosome 28 was generated by randomly inserting 10,000 SNPs into ARS-UCD1.2 chromosome 28 using the package SimuG [162]. A deep learning neural network based Nanopore read simulator, Deepsim [163], was used to simulate the Oxford Nanopore reads. The package is able to simulate Nanopore reads using a pore model which models the expected current signal when given a reference genome input [163]. Both the reference and mutated chromosome 28 were used as the reference genome input for the pore model in order to simulate a heterozygous diploid. The default base caller Albacore (version 2.3.1) was used to base call the raw current signal in the FAST5 files and generate the final reads in fastq format. An exponential read length distribution, used to simulate reads in humans [163], was used. The error profile of the simulated reads closely resembled that of reads generated with the MinION from cattle (Table S1, Figures S1–S3) and summary statistics were generated using NanoStat and NanoPlot [164]. To simulate a realistic on-farm sequencing preparation a mean read length of 2000 bp was used and various coverages (2 ×, 4 ×, 6 ×, 8 ×, 10 ×, 12 × and 20 ×) were simulated. The simulated Nanopore reads were mapped back to the original ARS-UCD1.2 chromosome 28. Samtools Mpileup [165] and BCFtools call [166] were used to produce VCF files with allele counts at each SNP loci. The individual base quality threshold (Q) was varied between 5, 7, 10 and the default 13 in the mpileup command. The probability of SNP discovery (P) in the BCFtools call command was set to 1 to allow discovery of all variants. An R script was used to call genotypes based on observations of reference or alternate alleles from the VCF file (Figure 4; Table S2). All heterozygous calls were categorised as either accurate or false positives (Figure 4) based on the observed allele matching the known substituted allele. This accounted for false positive heterozygous calls, caused by sequencing errors at the loci of interest.

An additional 10,000 base positions on chromosome 28 were also genotype called from each alignment as controls. All of these 10,000 positions were homozygous for the ARS-UCD1.2 reference allele. These positions were examined to determine the effect of sequencing errors on homozygous genotypes. The same genotype calling methods and parameters were used.

## 4. Results and Discussion

### 4.1. Heterozygous Positions

**Figure 5:**
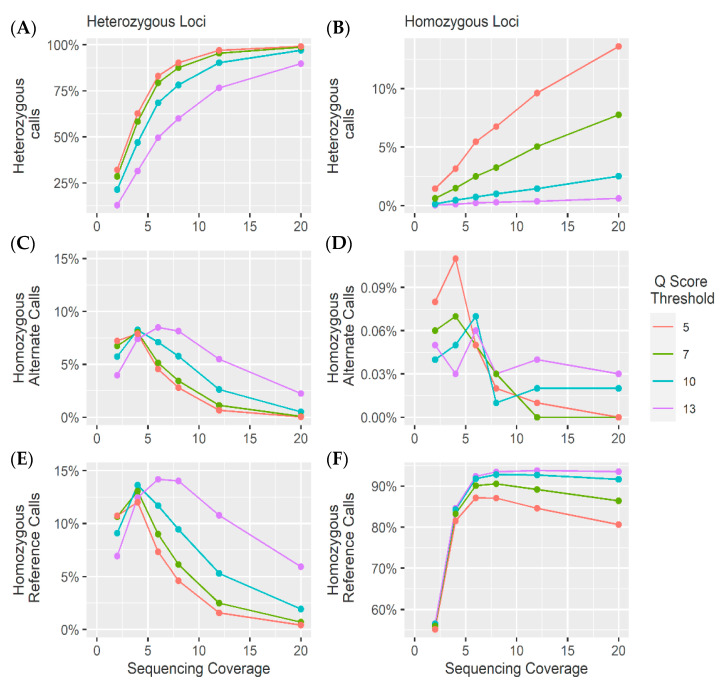
Genotype calling results from the 10,000 heterozygous loci (left) and 10,000 homozygous sites (right) at different sequencing coverages. (**A**) The percentage of SNPs correctly identified (heterozygous). (**B**) The percentage of false heterozygous alternate calls. (**C**) The percentage of false homozygous reference calls. (**D**) The percentage of false homozygous alternate calls. (**E**) The percentage of false homozygous reference calls. (**F**) The percentage of accurate homozygous reference calls.

**Figure 6:**
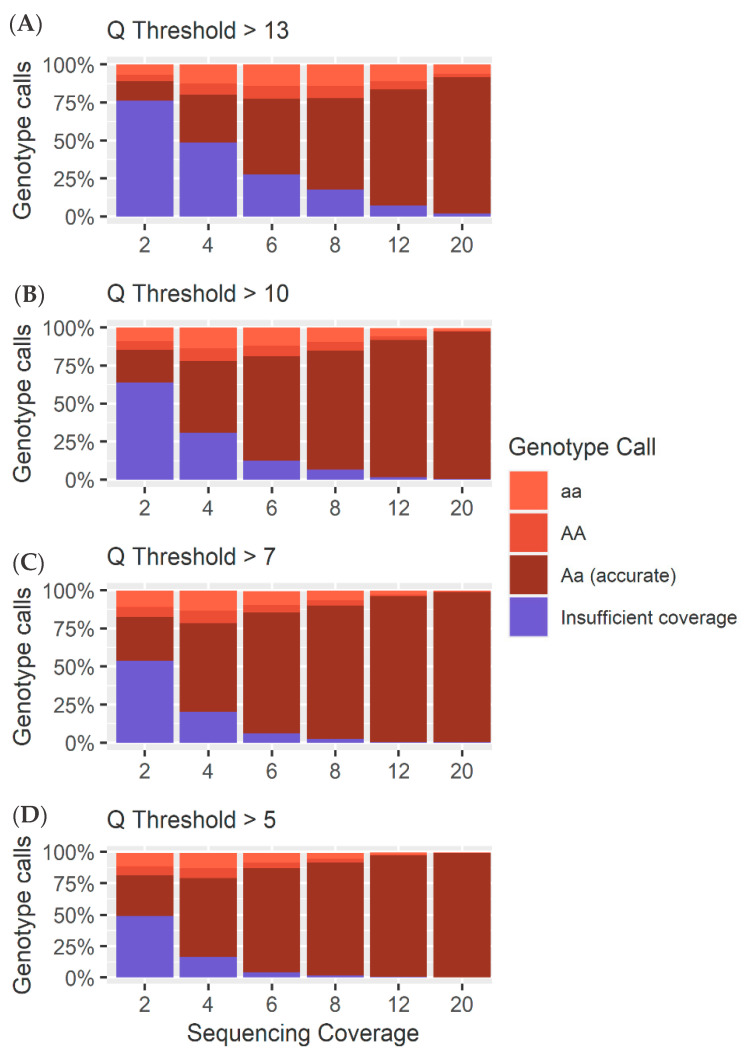
(**A**) A breakdown of the different calls (Table S2) for each sequencing coverage at a Q threshold of 13 for the 10,000 heterozygous loci. (**B**) A breakdown of the different calls (Table S2) for each sequencing coverage at a Q threshold of 10 for the 10,000 heterozygous loci. (**C**) A breakdown of the different calls (Table S2) for each sequencing coverage at a Q threshold of 7 for the 10,000 heterozygous loci. (**D**) A breakdown of the different calls (Table S2) for each sequencing coverage at a Q threshold of 5 for the 10,000 heterozygous loci.

**Figure 7:**
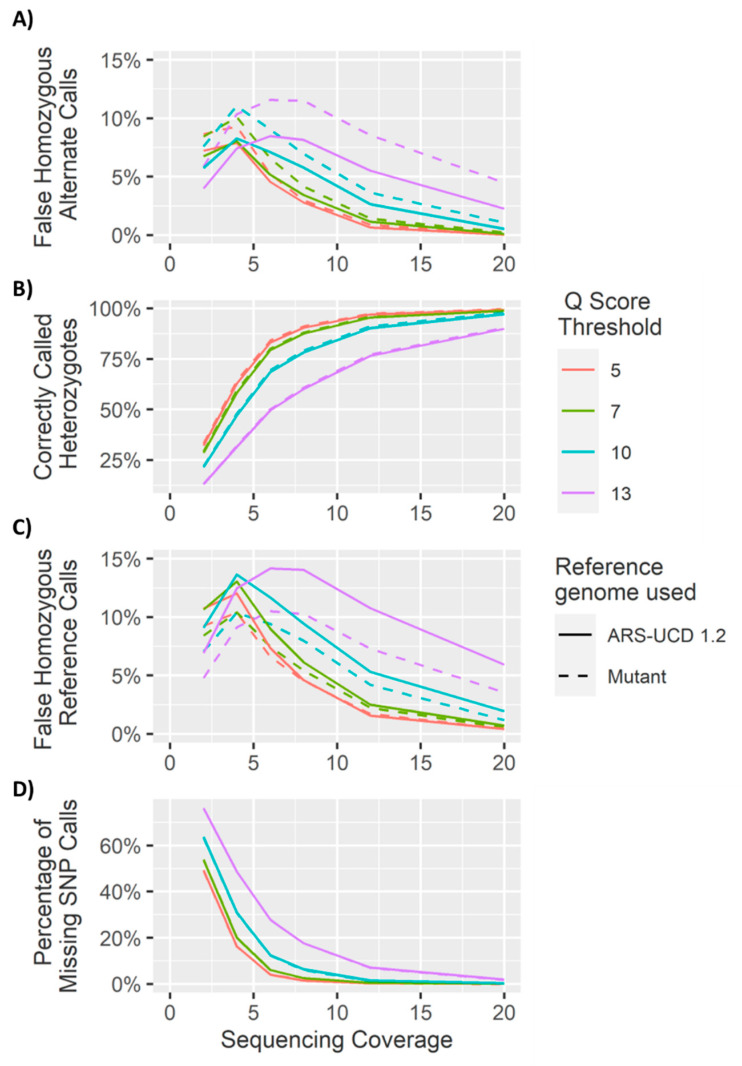
Genotype calling results from the alignments to chromosome 28 of ARS-UCD 1.2 and to the mutant chromosome at the 10,000 heterozygous sites. (**A**) The percentage of SNPs correctly identified (heterozygous). (**B**) The percentage of false homozygous alternate calls. (**C**) The percentage of false homozygous reference calls. (**D**) The percentage of SNP loci with coverage less than 2×.

The *Q* score threshold did not have a significant effect (*p* > 0.01) on the overall number of SNPs called (Figure 5), but did have a significant effect on the percentage of loci called correctly as heterozygous (*p* < 0.01), while sequencing coverage had a significant effect on both the overall number of SNPs called and the number of SNPs called correctly (*p* < 0.01). Accuracies of 85% were achieved with coverage as low as 6× and a *Q* score threshold of 5. The number of incorrect homozygous reference genotype calls was consistently higher than incorrect homozygous alternate calls (Figure 5 and Figure 6). This was thought to be an artifact of training and mapping to the reference genome ARS-UCD 1.2. Therefore, the same reads were mapped to the mutant chromosome where the proportion of homozygous reference to homozygous alternate alleles was more even (Figure 7 and Figure S4). This suggests that the bias between homozygous reference and homozygous alternate calls is related to the mapping of reads to a particular reference genome.

### 4.2. Homozygous Positions

**Figure 8:**
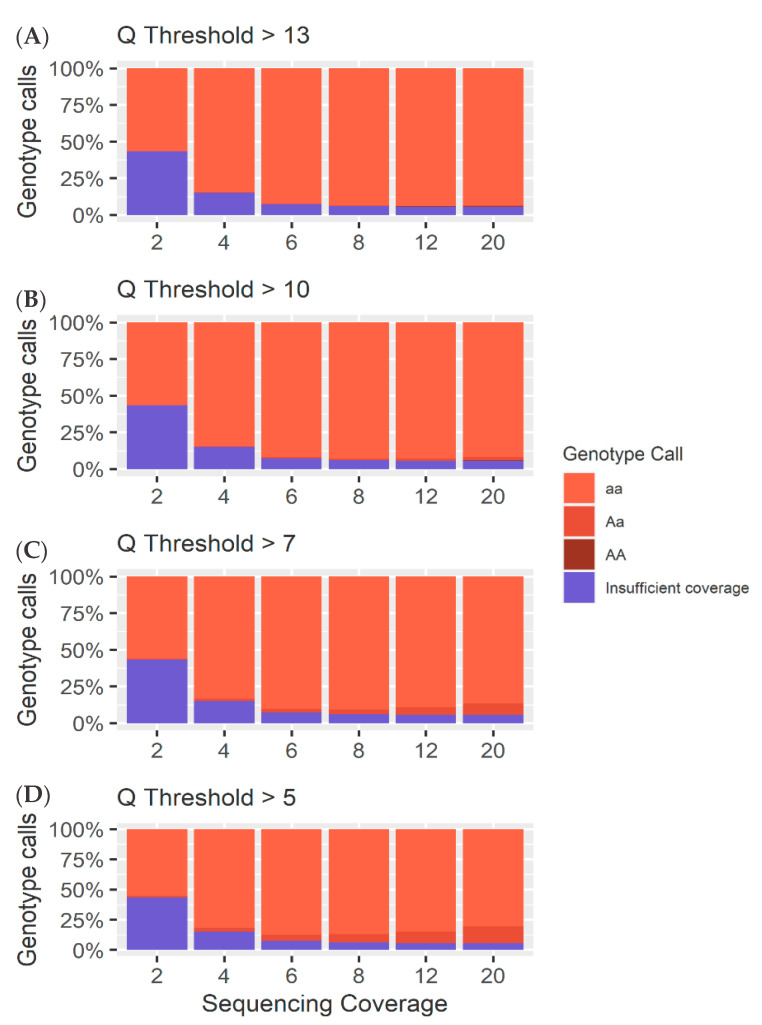
(**A**) A breakdown of the different calls (Table S2) for each sequencing coverage at a Q threshold of 13 for the 10,000 homozygous loci. (**B**) A breakdown of the different calls (Table S2) for each sequencing coverage at a Q threshold of 10 for the 10,000 homozygous loci. (**C**) A breakdown of the different calls (Table S2) for each sequencing coverage at a Q threshold of 7 for the 10,000 homozygous loci. (**D**) A breakdown of the different calls (Table S2) for each sequencing coverage at a Q threshold of 5 for the 10,000 homozygous loci.

Analysis of the genotype calls at the 10,000 homozygous loci demonstrated greater accuracy at lower coverages. However, at higher coverages, the method used to call heterozygotes meant there was an increase in heterozygous calls as a result of sequencing errors (Figure 5, Figure 6, Figure 7 and Figure 8). As coverage increased, the likelihood of at least one observation of an alternate allele from a sequencing error increased, consequently increasing the number of false heterozygous calls. The *Q* score threshold also had a significant effect (*p* < 0.01) on the number of false heterozygous calls, with a *Q* score threshold of 5 having far more false heterozygous calls than other *Q* scores. This is likely a result of the sequencing errors having a low base quality and therefore more sequencing errors being called with more relaxed *Q* score cut offs. Accuracies of almost 95% were achieved for these loci at coverages as low as 6 ×, very similar to results reported by Malmberg, et al. [138]. The percentage of homozygous alternate calls remained extremely low across all sequencing coverages with a maximum of just over 0.1% at 4 × sequencing coverage. These results suggest that a low Q threshold is favourable for heterozygous calling. However, a more stringent Q threshold is necessary to prevent false heterozygous calls at homozygous loci. False homozygous calls at low-coverage heterozygous loci are a considerable limitation of low-coverage genotyping [167]. A number of studies have proposed the maximum likelihood (ML) method as a solution [166,168]. The use of allele dosage/ probability based genotyping, as is often implemented when using Illumina data [169] would likely decrease the number of false homozygous genotypes. Wang, et al. [167] demonstrated that this method for genotype correction in GBS data increased the rate of correct genotypes as well as the accuracy of genomic prediction. However, SNP array genotyping was more accurate than the corrected GBS genotypes for coverages below 10 × [166]. Alternatively, Dodds, et al. [161] describe a method for calculating unbiased estimates of relatedness by using only SNPs with genotype calls in both individuals. They refer to this method as kinship using GBS with depth adjustment (KGD) [161]. Despite being developed specifically for use in GBS data, the similarities between GBS and the crush-side genotyping method proposed here suggest KGD could adapted for crush-side genotyping.

Overall, these simulations have highlighted genotype calling using Nanopore data is possible in both homozygous and heterozygous populations. However, a more refined method for genotype calling is required to increase the accuracy of genotype calling in highly heterozygous populations. At high coverage (>6×) the error rates reported here for heterozygous and homozygous positions appear to be within the tolerable threshold of genomic prediction given imputation error rates of 5.5–0.9% are commonly reported [161,170,171,172,173,174]. Validation of the results reported here on real datasets is still required. However these results provide preliminary evidence that Oxford Nanopore sequencing has potential for on-farm SNP genotyping for current GBLUP predictions. Importantly, even read lengths that are relatively short for MinION data did not result in decreased SNP calling accuracy.

## 5. Conclusions

The MinION has opened new applications for sequencing technology as well as providing benefits to current sequencing applications. Its portability, read length and real-time base calling capabilities have cemented it as a new rapid diagnostic tool, particularly in humans, despite the high error rates. A number of studies have also now demonstrated potential applications in agriculture as a diagnostic tool for disease and antibiotic resistance [38,42,53]. In particular, O’Donnell, et al. [38] demonstrated the effective use of the MinION to monitor the 2019 outbreak of ASF and developed a real-time rapid diagnostic tool, ASF-FAST. Furthermore, the MinION provides enormous benefits in characterising complex genomic regions for genome assemblies. The snow sheep, giant redwood and giant sequoia genome assemblies have all demonstrated increased contiguity as a result of using long Nanopore reads [5,6,7]. More accurate genome assemblies will also allow the MinION to be used effectively in the characterisation and calling of structural variants to increase our understanding of genetic variation [110], which is of particular interest in livestock. This may help quantify some of the missing heritability in a number of common diseases and economically desirable traits. Additionally, the ability to directly capture base modifications in real time could help to one day incorporate methylation and epigenetics into genomic predictions. With strong evidence suggesting a correlation between premature ageing and methylation [130,131,132], we see carcass ossification as an obvious application of this.

With the ability to rapidly genotype both SNPs and SVs, we propose the use of the MinION for crush-side genotyping in Australia’s Northern cattle industry to deliver on-farm genomic prediction. The capacity to sequence, map and process reads in real time means that crush-side genotyping can provide rapid genomic prediction to industries where laboratory turnaround time is a major limitation. The power to call causative mutations rather than SNPs sharing LD and rapidly incorporate new markers using a script rather than developing entirely new SNP arrays will have significant merit as new causal mutations are discovered.

Our preliminary in silico investigation assessed the feasibility of low-coverage SNP genotyping in a heterozygous population using simulated Nanopore data. An artificial diploid of *B. taurus* chromosome 28 with 10,000 SNPs was created using the package SimuG and filtering and calling parameters were altered to investigate genotyping accuracies. The results suggested that coverages as low as 6× can produce genotyping accuracies greater than 85%. These results provide preliminary evidence that MinION sequencing has the potential to be used for rapid on-farm crush-side genotyping for genomic prediction in Australia’s northern beef industry.

## Supplementary Materials

The following are available online at https://www.mdpi.com/2073-4425/11/12/1478/s1, Figure S1: Read length histogram for real and simulated Nanopore data, Figure S2: Average read quality versus aligned length for real and simulated Nanopore data, Figure S3: Average read quality versus read length for real and simulated Nanopore data, Figure S4: Comparison of genotype calls for heterozygous positions aligned to different reference genomes, Table S1: Comparison between the simulated and real Nanopore read statistics, Table S2: Definitions of Genotype Calls.
