# Supplementary material for: The Future of Livestock Management: A Review of Real-Time Portable Sequencing Applied to Livestock
Source: Genes (Basel). 2020 Dec 9;11(12):1478. doi: 10.3390/genes11121478 (PMC7763041; doi:10.3390/genes11121478)
Supplement: Supplementary file 1 [file genes-11-01478-s001.zip › Supplementary File 1.docx]

**Supplementary File 1**

**Table S1.** Comparison between the simulated and real Nanopore read statistics. Comparison of summary read statistics for the simulated reads and real reads from a tail hair sample run on an R9.4 flow cell and base called using Guppy 3.4.

| **Parameter** | **Real Reads** | **20 x Simulation Reads** |
| --- | --- | --- |
| Mean Read Length | 1,499.1 | 2,913.20 |
| Mean Read Quality | 12.9 | 10.6 |
| Median Read Quality | 12.9 | 10.6 |
| Read length N50 | 2,216.0 | 3,550.00 |
| > Q5 | 100 | 100 |
| > Q7 | 100 | 99.99 |
| > Q10 | 92 | 96.3 |
| > Q15 | 13.9 | 0 |

A)

B)


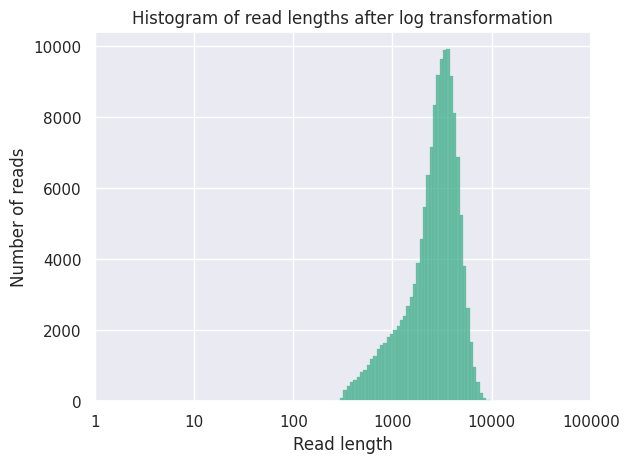


**Figure S1.** Read length histogram for real and simulated Nanopore data. **A**) Histogram of read length for real data generated using the minION (flow cell R9.4.1) and base called with Albacore. **B**) Histogram of read length for the reads simulated using DeepSim.


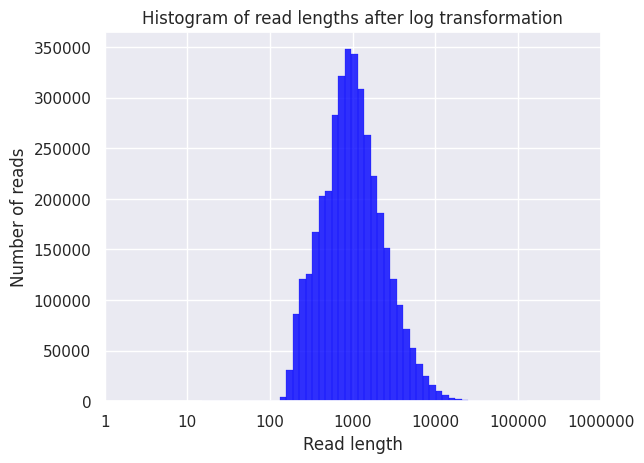


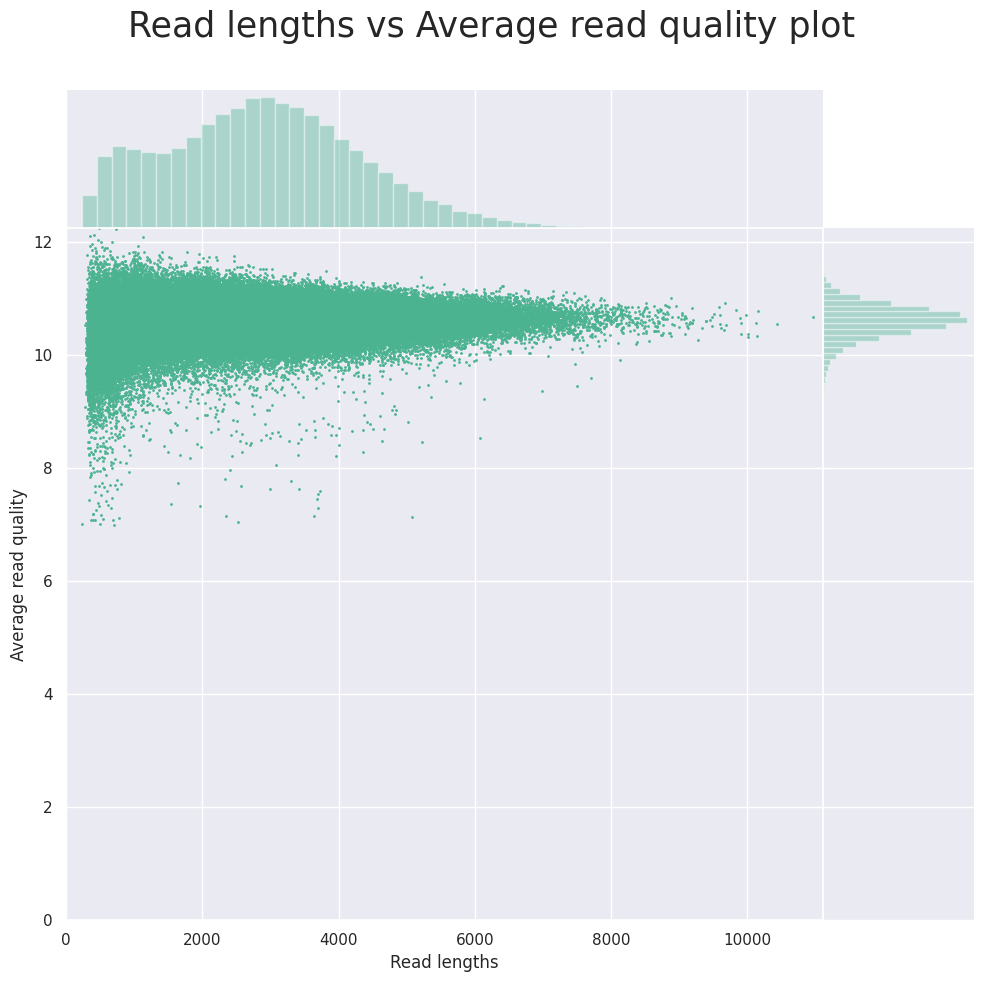

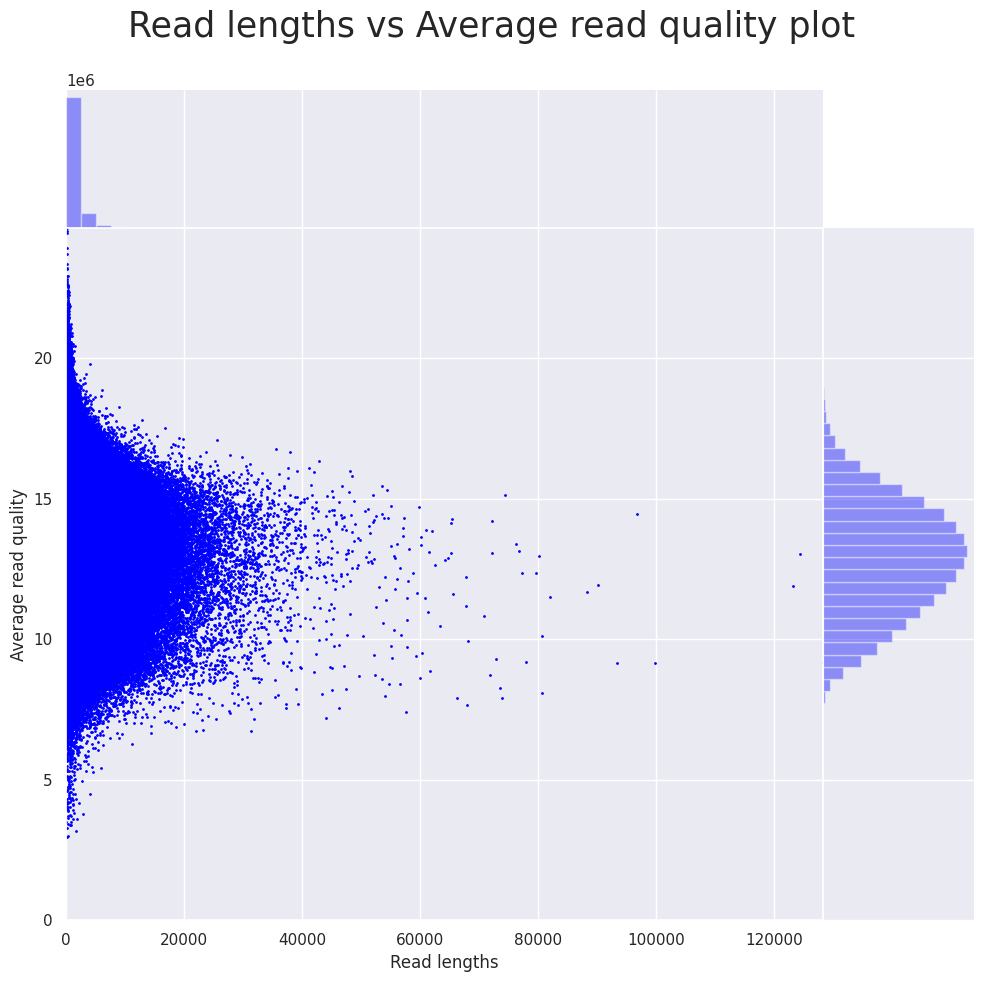


**Figure S2.** Average read quality versus aligned length for real and simulated Nanopore data. **A**) Average read quality versus aligned read length for real data generated using the minION (flow cell R9.4.1) and base called with Albacore. **B**) Average read quality versus aligned read length for the reads simulated using DeepSim.

B)

A)


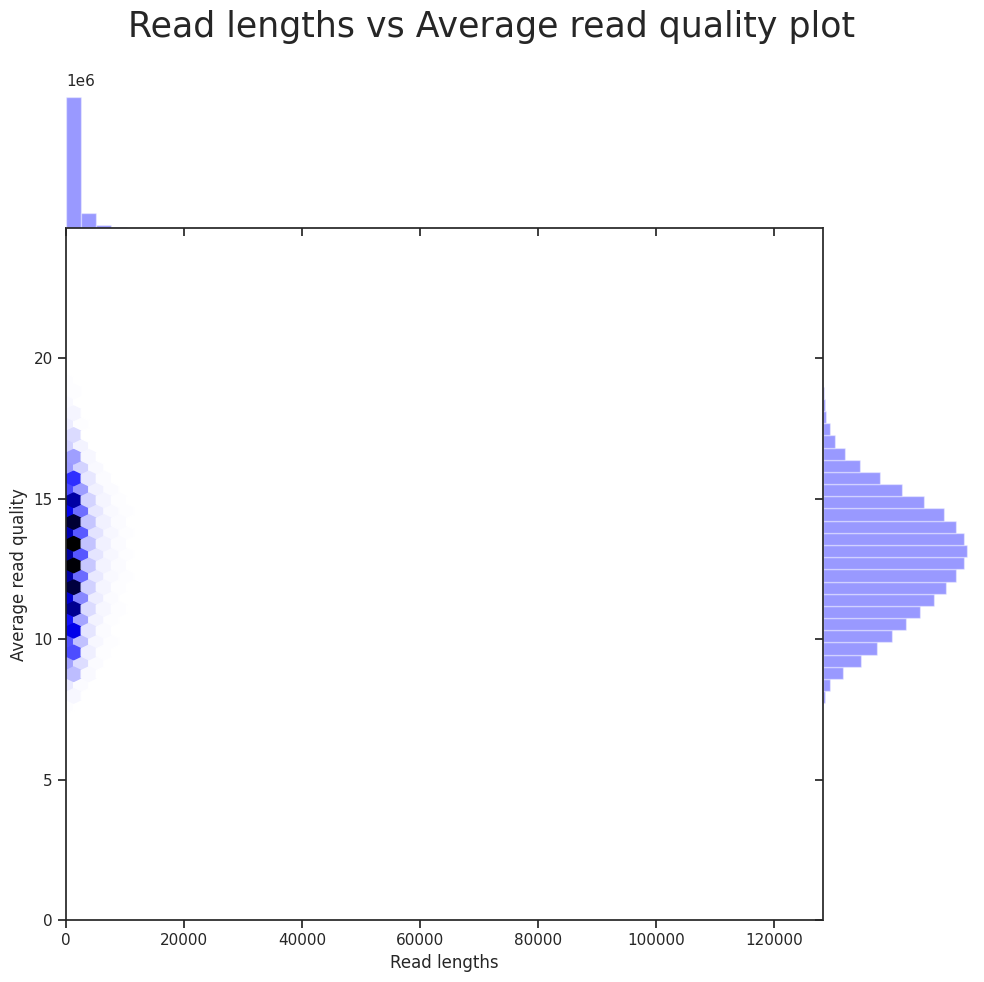

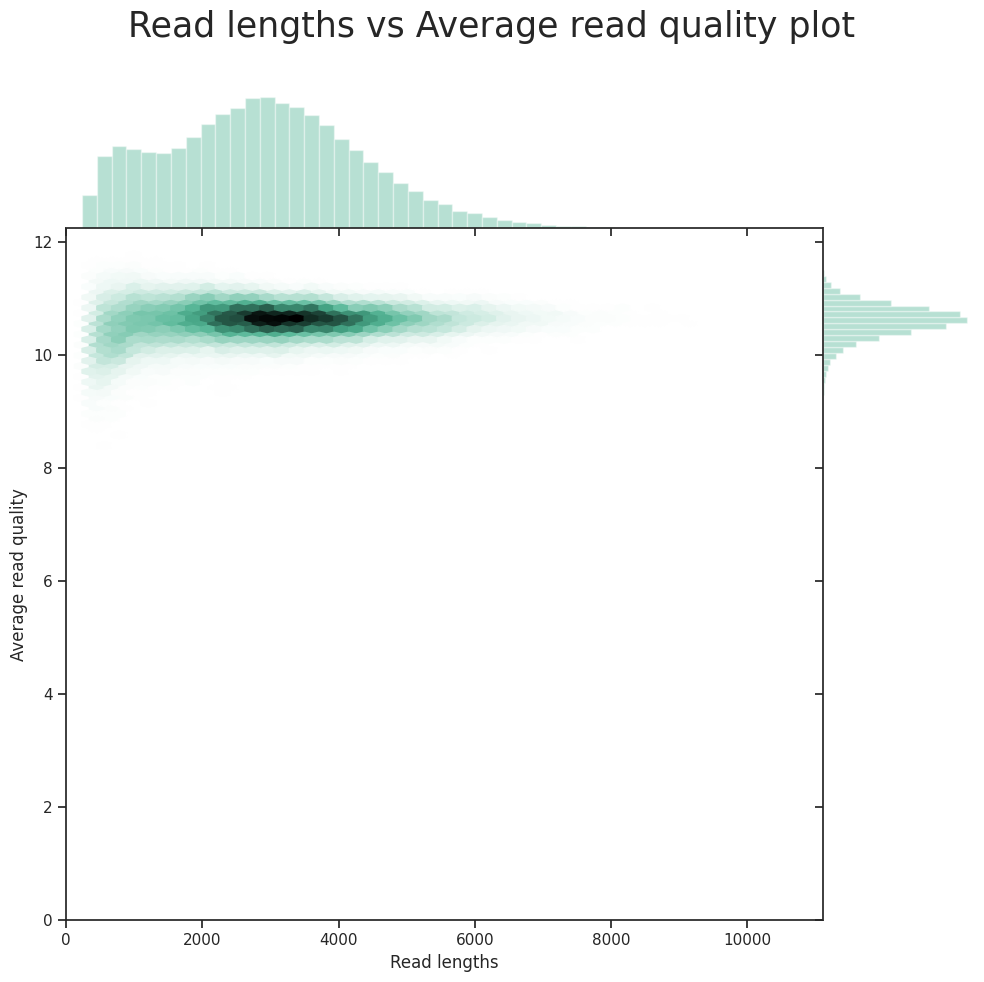


**Figure S3**. Average read quality versus read length for real and simulated Nanopore data. **A**) Average read quality versus read length for real data generated using the minION (flow cell R9.4.1) and base called with Albacore. **B**) Average read quality versus read length for the reads simulated using DeepSim.

B)

A)
