# Supplementary material for: The Future of Livestock Management: A Review of Real-Time Portable Sequencing Applied to Livestock
Source: Genes (Basel). 2020 Dec 9;11(12):1478. doi: 10.3390/genes11121478 (PMC7763041; doi:10.3390/genes11121478)
Supplement: Supplementary file 1 [file genes-11-01478-s001.zip › Supplementary File 2.docx]

**Table S2.** Definitions of Genotype Calls.

The following definitions were used in the assigning of genotype calls from the observations of alleles:

Insufficient Coverage:

A loci was labelled as having insufficient coverage if at that loci there were less than 2 observations of an allele that passed the phred base threshold stipulated.

Heterozygous Genotypes (Aa)

A loci was called heterozygous where at that loci there was an observation of at least one reference and one alternate allele that passed the phred base threshold stipulated.

Homozygous Reference Genotypes (aa)

A loci was called homozygous reference where at that loci there was an observation of at least two reference alleles and no observations of alternate alleles that passed the phred base threshold stipulated.

Homozygous Alternate Genotypes (AA)

A loci was called homozygous alternate where at that loci there was an observation of at least two alternate alleles and no observations of reference alleles that passed the phred base threshold stipulated.
