# Supplementary material for: The Future of Livestock Management: A Review of Real-Time Portable Sequencing Applied to Livestock
Source: Genes (Basel). 2020 Dec 9;11(12):1478. doi: 10.3390/genes11121478 (PMC7763041; doi:10.3390/genes11121478)
Supplement: Supplementary file 1 [file genes-11-01478-s001.zip › Supplementary File 3.docx]

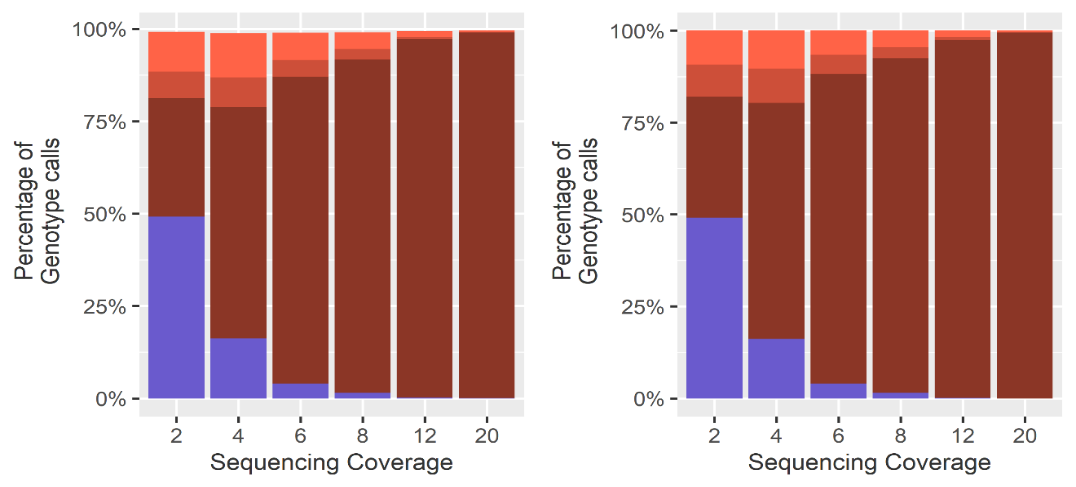

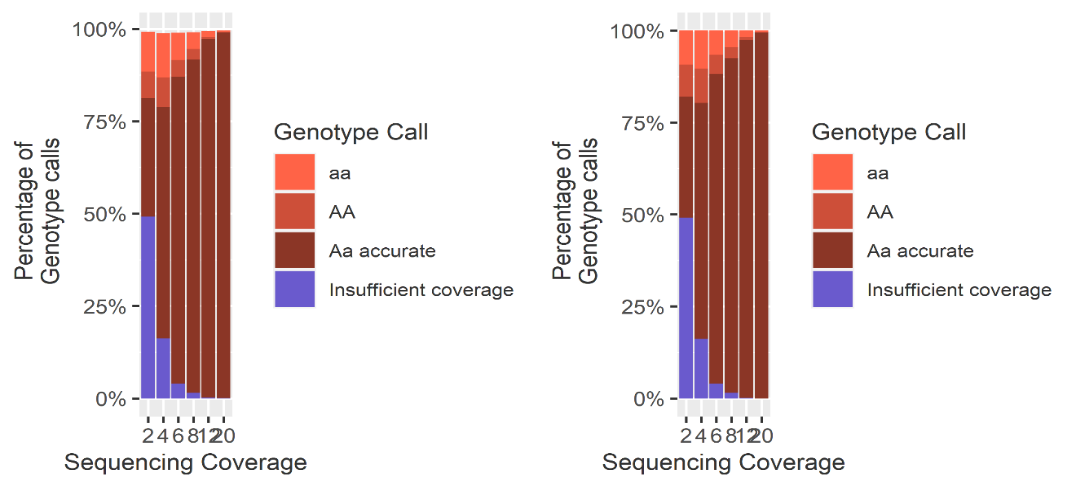


**A)**

**Figure S4.** Comparison of genotype calls for heterozygous positions aligned to different reference genomes. **A**) Percentage of genotype calls for the heterozygous positions aligned to ARS-UCD 1.2 for a Q threshold of 5. **B**) Percentage of genotype calls for the heterozygous positions aligned to the mutant chromosome for a Q threshold of 5.

**B)**
